# Circulating cytokine levels in systemic sclerosis related interstitial lung disease and idiopathic pulmonary fibrosis

**DOI:** 10.1038/s41598-023-31232-4

**Published:** 2023-04-24

**Authors:** Boyang Zheng, Kevin J. Keen, Marvin J. Fritzler, Christopher J. Ryerson, Pearce Wilcox, Beth A. Whalen, Basak Sahin, Iris Yao, James V. Dunne

**Affiliations:** 1 Division of Rheumatology, McGill University, Montreal, QC Canada; 2 Department of Medicine, University of British Columbia, Vancouver, BC Canada; 3 Centre for Heart Lung Innovation, Providence Research, St. Paul’s Hospital, University of British Columbia, Vancouver, BC Canada; 4 Department of Mathematics and Statistics, University of Northern British Columbia, Prince George, BC Canada; 5 Department of Medicine, Cumming School of Medicine, University of Calgary, Calgary, AB Canada; 6 Suite 330, 2184 West Broadway, Vancouver, BC V6K 2E1 Canada

**Keywords:** Immunology, Biomarkers, Rheumatology, Connective tissue diseases

## Abstract

Exploration of cytokine levels in systemic sclerosis-associated interstitial lung disease (SSc-ILD) and idiopathic pulmonary fibrosis (IPF) is needed to find common and diverse biomolecular pathways. Circulating levels of 87 cytokines were compared amongst 19 healthy controls and consecutive patients with SSc-ILD (n = 39), SSc without ILD (n = 29), and IPF (n = 17) recruited from a Canadian centre using a log-linear model adjusted for age, sex, baseline forced vital capacity (FVC), and immunosuppressive or anti-fibrotic treatment at time of sampling. Also examined was annualized change in FVC. Four cytokines had Holm’s corrected p-values less than 0.05. Eotaxin-1 levels were increased approximately two-fold in all patient categories compared to healthy controls. Interleukin-6 levels were eight-fold higher in all ILD categories compared to healthy controls. MIG/CXCL9 levels increased two-fold more in all but one patient category compared to healthy controls. Levels of a disintegrin and metalloproteinase with thrombospondin type 1 motif, member 13, (ADAMTS13) were lower for all categories of patients compared to controls. No substantial association was found for any of the cytokines with FVC change. Observed cytokine differences suggest both common and diverse pathways leading to pulmonary fibrosis. Further studies evaluating longitudinal change of these molecules would be informative.

## Introduction

Systemic sclerosis (SSc) is an autoimmune fibrosing disorder that can affect the lungs, leading to interstitial lung disease (ILD) in approximately 30–50% of patients^1,2^. ILD is a morbid condition that can also be caused by multiple genetic and environmental factors, with isolated idiopathic pulmonary fibrosis (IPF) being a frequent subtype with a particularly poor outcome. While biologic pathways are poorly understood in both SSc-ILD and IPF, some interactions between adaptive and innate immune responses with fibrotic pathways could be hypothesized to be common drivers of both^3^.

Our primary objective was to compare the levels of 87 circulating cytokines sampled from plasma in healthy controls to patients with SSc without ILD, SSc-ILD, or IPF. Our secondary objective was to determine whether any of the differentially expressed cytokines were associated with subsequent ILD progression as determined by decline in forced vital capacity (FVC) or diffusing capacity of the lung for carbon monoxide (DLCO).

## Methods

### Participants and study design

Healthy participants (without a diagnosis of SSc, other connective tissue disease, diabetes, chronic lung disease, or a history of smoking; were not on medications for heart disease or hypertension, and did not have a history of myocardial infarction) were recruited by the Scleroderma Association of British Columbia (SABC) Research Program through public outreach. Consecutive SSc patients with and without ILD were recruited from SABC Research Program Registry (2011–2020). IPF patients were recruited from one participating site of the Canadian Registry for Pulmonary Fibrosis (2017–2020)^4^. All patients with SSc fulfilled the 2013 EULAR/ACR classification criteria^5^. Patients with SSc were classified as diffuse cutaneous SSc (dcSSc) if they had skin involvement extending proximally from the elbows or knees and as limited cutaneous SSc (lcSSc) if they had skin involvement distal to the elbows and knees. All ILD diagnoses were made according to established clinical practice guidelines based on review of high resolution computed tomography (HRCT) scans and lung biopsy specimens when performed^6,7^. ILD progression was assessed by examining the longitudinal course of percent-predicted FVC and DLCO on serial pulmonary function tests (PFTs) over the 2 years following the date of phlebotomy, with an annualized FVC decline ≥ 10% considered to be clinically substantial progression. PFTs were performed typically every 3–6 months as clinically indicated. Baseline covariates included age, sex, cumulative smoking pack years, ever-smoking status, and the presence of treatment with anti-fibrotic (nintedanib or pirfenidone) or immunosuppressive (mycophenolate mofetil, azathioprine, rituximab, or cyclophosphamide) medication at the time of phlebotomy.

### Cytokine testing

Whole blood samples collected in dipotassium ethylenediaminetetraacetic acid (K2 EDTA) vaccutainers were centrifuged at 1900 g for 15 min at 4 °C within an hour of collection. Plasma was obtained and 500 μL aliquots were stored in RNAse/DNase free cryovials at − 80 C before being transported on dry ice for testing. Commercial multiplex panels (MilliporeSigma) were used to assay 87 biomarkers (Human Cytokine/Chemokine 71-Plex Discovery Assay® Array (HD71); Human Soluble Cytokine Receptor 14-Plex Discovery Assay® Array (HDSCR14); ADAMTS13 and serum amyloid A: Eve Technologies, Calgary, Canada). The complete list of cytokines is provided in Supplementary Table S1. Quality control (QC) was performed with each assay session in accordance with the Clinical Laboratory Improvement Amendments (CLIA) using kits supplied by the manufacturer according to their instructions (MilliporeSigma). Control charts were used with a moving range and applied Westgard rules to assess QC performance. The accuracy and precision of the assays were validated in accordance with CLIA guidelines. Cytokine levels measured in clinical samples have been demonstrated to be stable and reproducible over a 16-day period of specimen collection. Cytokine measurements were not normalized.

### Statistical analysis

The primary analysis compared log-transformed cytokine levels between healthy controls, IPF, dcSSc with and without ILD, and limited SSc (lcSSc) with and without ILD using multivariable linear regression models. Healthy controls were the reference group and all were adjusted for age and sex interaction, disease duration, ever smoking, and the presence of anti-fibrotic or immunosuppressive treatments, separately, as of the sampling date. The differentially expressed cytokines identified were then assessed for association with FVC decline and DLCO decline in multivariable linear regression models adjusted for age, sex, smoking status, treatment presence, and baseline FVC. Statistical significance was corrected for multiplicity of testing using Holm’s method for all models^8^. Analyses were performed using R software (version 4.2.2)^9^. A post-hoc power analysis for the primary findings at the 5% level of significance was performed with PASS software (version 22.0.3)^10^ in an analysis-of-covariance model with 7 covariates with group log-folds, proportion of variance explained by the multiple regression model, and residual error as estimated by R software. Point and interval estimates of effect size for the main effect of differences amongst the disease types and healthy controls were performed by R software.

### Ethics approval and consent to participate

This study was conducted according to the ethical principles of the Declaration of Helsinki. Ethics approval was obtained from the Research Ethics Boards of Providence Health Care (which also acts on behalf of The University of British Columbia) and The University of Northern British Columbia. Voluntary informed consent was obtained from all participants.

## Results

### Baseline characteristics in participants

Blood samples were obtained from 19 healthy controls, 17 patients with IPF, 16 with dcSSc-ILD, 11 with dcSSc and no ILD, 23 with lcSSc-ILD, and 18 with lcSSc and no ILD (Table 1). The statistically and practically significant differences in baseline characteristics amongst the participant categories were as expected for age (*p* < 0.0001), disease duration (*p* < 0.0001), ever smoker (*p* = 0.0020), pack years (*p* = 0.0003), antifibrotic treatment (*p* < 0.0001), and immunosuppressive treatment (*p* = 0.0003). These results corroborate the conventional wisdom that compared to patients with SSc, patients with IPF are mostly older males with the past history of being heavy smokers with a shorter life expectancy after diagnosis compared to patients with any form of SSc. In this sample, 41% of the patients with IPF were receiving antifibrotic treatment at time of phlebotomy compared to none of the patients with SSc. None of the patients with IPF were receiving immunosuppressive treatment. The differences amongst the patient groups in this sample were otherwise statistically insignificant with respect to baseline FVC percent-predicted (*p* = 0.0753), baseline DLCO percent-predicted (*p* = 0.0588), annualized FVC percent-predicted annualized change (*p* = 0.1365), and annualized DLCO percent-predicted annualized change (*p* = 0.5252).

**Table 1:** Baseline participant characteristics.

	Healthy Control (n = 19)	IPF (n = 17)	dcSSc ILD(n = 16)	dcSSc No ILD (n = 11)	lcSSc ILD (n = 23)	lcSSc No ILD (n = 18)	*p*-value
Age	51 ± 19	73 ± 7	52 ± 14	54 ± 14	58 ± 11	58 ± 10	< 0.0001
Male Count (%)	6 (32%)	12 (71%)	8 (50%)	3 (27%)	4 (17%)	1 (6%)	
Disease duration	NA	1.8 ± 2.1	3.6 ± 5.1	7.6 ± 10.2	7.9 ± 8.7	11.6 ± 7.1	< 0.0001
Ever smoker (%)	3 (16)	14 (82)	8 (50)	4 (36)	12 (52)	6 (33)	0.0020
Pack years [range]	0[0, 1]	22[0, 90]	5[0,30]	4[0, 38]	12[0,51]	6[0,40]	0.0003
Baseline FVC % (sample size)	NA	85 ± 20 (17)	75 ± 17 (12)	113 ± 7 (3)	83 ± 25 (16)	88 ± 7 (2)	0.0753
Baseline DLCO % (sample size)	NA	49 ± 11 (13)	49 ± 12 (12)	79 ± 12 (3)	50 ± 19 (16)	63 ± 3 (2)	0.0588
Antifibrotic Treatment (%)	0 (0%)	7 (41%)	0 (0%)	0 (0%)	0 (0%)	0 (0%)	< 0.0001
Immunosuppressive Treatment (%)	0 (0%)	0 (0%)	6 (38%)	1 (10%)	9 (39%)	0 (0%)	0.0003
Annualized FVC % Change (sample size)	NA	− 6.2 ± 13.6 (17)	− 3.1 ± 9.2 (14)	− 0.2 ± 4.4 (3)	− 1.0 ± 7.6 (20)	NA	0.1365
Annualized DLCO % Change (sample size)	NA	− 7.8 ± 18.6 (14)	− 1.2 ± 5.5 (10)	0.8 ± 3.2 (3)	− 0.4 ± 6.8 (14)	NA	0.5252

Disease duration is defined as time of ILD first seen on HRCT in IPF and time from first non-Raynaud’s phenomenon in SSc with and without ILD. Antifibrotic treatment includes nintedanib or pirfenidone. Immunosuppressive treatment includes azathioprine, cyclophosphamide, mycophenolate mofetil, or rituximab. Mean ± SD reported for continuous variables except pack years for which the mean and range is reported. Count and percentage proportion reported for dichotomous variables. The *p*-values computed for the continuous variables are by the nonparametric Kruskal–Wallis for no shift in location for available groups. The *p*-values for the categorical variables are by Fisher’s exact test.

### Cytokine associations with ILD presence

**Figure 1:**
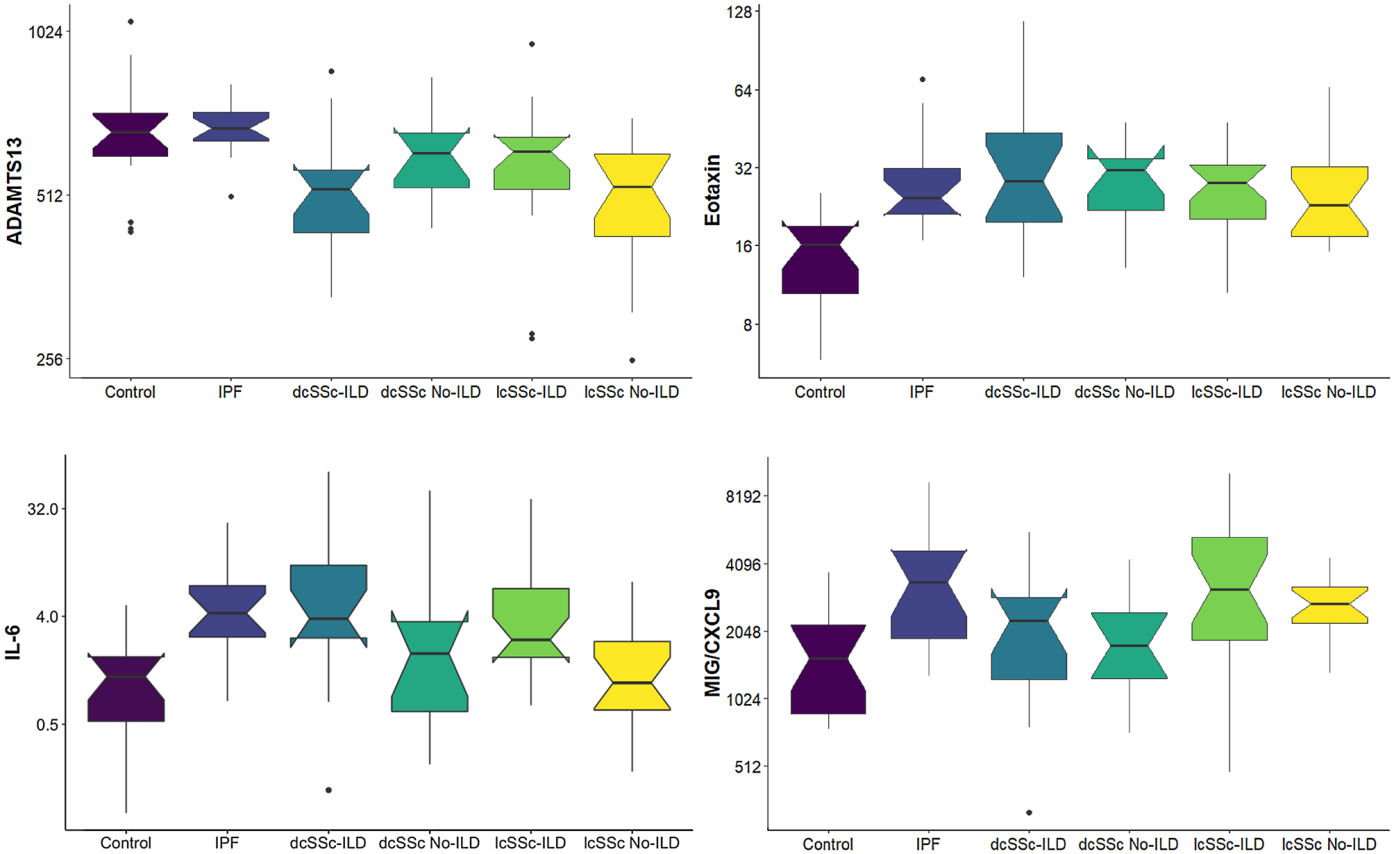
Notched box plots of identified cytokine differences between disease groups. All cytokine levels are shown on a log scale.

Out of 87 biomarkers assessed, 4 were substantially different amongst the 6 different categories of participants with Holm’s *p*-values adjusted for multiple testing (Table 2 and Fig. 1) in models with baseline covariates for age, disease duration, ever-smoking status, antifibrotic medication (nintedanib or pirfenidone) and immunosuppresive medication (azathioprine, cyclosphosphamide, mycophenolate mofetil or rituximab) at time of phlebotomy. A post hoc analysis showed the power for each of the four cytokines to be between 0.9838 and 0.9999 using PASS software in an analysis-of-covariance model with 7 covariates with group log-folds, proportion of variance explained by the multiple regression model, and residual error as estimated from the data by R software (Table 2). Effect size was assessed by computing the ${\eta }_{p}^{2}$ statistic. Rule-of-thumb lower cut-offs for small, medium, and large effect sizes are 0.01, 0.06, and 0.14, respectively. The bootstrapped 95% confidence intervals for all four cytokines include the lower cut-offs for both medium and large effect sizes with point estimates of effect size ranging from 0.22 to 0.28 for the four cytokines (Table 2).

**Table 2:** Log-fold changes in cytokine levels by disease group compared to control.

Cytokine	Disease	Log-fold Estimate	2.5 Percentile	97.5 Percentile	Holm’s *p*-value	Post Hoc Power	Effect Size ${{\varvec{\eta}}}_{{\varvec{p}}}^{2}$ 95% CI
ADAMTS13	IPF	0.090	− 0.237	0.418	0.0363	0.9998	0.22
	dcSSc ILD	− 0.026	− 0.536	0.014			(0.09, 0.63)
	dcSSc NoILD	− 0.068	− 0.360	0.224			
	lcSSc ILD	− 0.400	− 0.124	0.150			
	lcSSc NoILD	− 0.383	− 0.662	− 0.104			
Eotaxin-1	IPF	0.753	0.185	1.321	0.0011	0.9999	0.28
	dcSSc ILD	1.088	0.610	1.566			(0.13, 0.70)
	dcSSc NoILD	0.829	0.320	1.338			
	lcSSc ILD	0.805	0.330	1.281			
	lcSSc NoILD	0.617	0.133	1.101			
IL-6	IPF	1.879	0.398	3.359	0.0072	0.9998	0.25
	dcSSc ILD	2.259	1.013	3.505			(0.11, 0.67)
	dcSSc NoILD	0.937	− 0.390	2.263			
	lcSSc ILD	1.977	0.738	3.217			
	lcSSc NoILD	0.349	− 0.913	1.610			
MIG/CXCL9	IPF	0.468	− 0.298	1.233	0.0368	0.9838	0.22
	dcSSc ILD	0.524	− 0.120	1.168			(0.09, 0.63)
	dcSSc NoILD	0.264	− 0.421	0.950			
	lcSSc ILD	1.056	0.415	1.696			
	lcSSc NoILD	0.707	0.055	1.359			

Estimates, confidence limits, Holm’s *p*-value, and effect size with bootstrapped 95% confidence interval (CI) are from a multiple regression linear model using R software of the base-2 logarithm of each cytokine level from peripheral blood for differences among categories with: continuous covariates of age and disease duration; categorical covariates of ever smoking, sex, antifibrotic medication (nintedanib or pirfenidone), anti-autoimmune medication (azathioprine, cyclophosphamide, mycophenolate mofetil, or rituximab); and interaction between age and sex. The post hoc power for a test at the 5% level of significance was estimated using PASS software in an analysis-of-covariance model with 7 covariates with group log-folds, proportion of variance explained by the multiple regression model, and residual error as estimated by R software.

ADAMTS13 was different amongst the 6 categories (Holm’s *p* = 0.0363) with all patients with SSc having lower levels compared to healthy controls and patients with IPF. ADAMTS13 levels, however, in patients with the mildest form of disease, lcSSc, were the lowest. Eotaxin-1 was also different amongst the 6 categories (Holm’s *p* = 0.0011) with the fold-change not different from double for each category of patient compared to healthy controls. Interleukin 6 (IL-6) was different with all patient categories (Holm’s *p* = 0.0072) being elevated compared to healthy controls. Remarkably, there appears to be a substantial fold-change of 8 for patients with either IPF, dcSSc-ILD, and lcSSc-ILD compared to healthy controls, but no difference for patients either with dcSSc and no ILD or with lcSSc and no ILD. This suggests that higher plasma levels of IL-6 are a feature of ILD regardless of whether it is primary or secondary to SSc, but not SSc without ILD. Levels of monokine induced by gamma interferon, or chemokine (C-X-C motif) ligand-9, (MIG/CXCL9) were statistically different among the six categories of participants. A remarkable fold doubling was observed for both groups of patients with lcSSc, either with or without ILD, compared to healthy controls, but no difference was observed for the other patient groups.

### Association of cytokines levels with change in FVC

Annualized decline in percent-predicted FVC in patients with IPF (median − 6.2, IQR 13.6) was not statistically different (*p* = 0.1692) compared to patients with SSc-ILD (median − 1.7, IQR 8.2). The association between the log cytokine levels and annualized rate of FVC decline was examined for each of the 4 differentially expressed cytokines in separate multiple linear regression models. None of these cytokines had a substantial or statistically significant association with longitudinal FVC change or progression defined by ≥ 10% decline in percent-predicted FVC after adjusting for baseline covariates (age, sex, smoking status, FVC, and the presence of disease specific treatment) after correcting for multiple testing in IPF, lcSSc-ILD, dcSSc-ILD, or the combined cohort of patients with ILD. Similarly, there were no substantial or statistically significant associations between these 4 cytokines and the rate of DLCO decline.

## Discussion

Using two large commercially available cytokine panels, 4 cytokine associations with SSc-ILD and IPF were identified after correction for multiplicity of tests by Holm’s method^8^. Differentially expressed cytokines ADAMTS13, Eotaxin-1, IL-6, and MIG/CXCL9 are involved in pathways mediating immune modulation or crosstalk among pro-fibrotic cells and have biological plausibility in SSc, SSc-ILD, and IPF. However, no predictors of ILD progression based on FVC or DLCO decline were identified among all 87 cytokines tested.

### Common cytokine profiles in SSc-ILD and IPF

Eotaxin-1 was elevated in IPF patients and SSc patients, with or without ILD, regardless of cutaneous form, compared to healthy controls. Eotaxin-1 is an eosinophil chemoattractant protein responsible for immune cell extravasation that is increased in allergic lung diseases^11^ and also bleomycin mouse models of pulmonary fibrosis^12^, inclusive of SSc-ILD. In bleomycin-induced lung fibrosis in mouse models, higher tissue levels of eotaxin-1 are associated with increased pulmonary infiltration of eosinophils and neutrophils and the production of profibrotic cytokines^12^. Elevations of serum eotaxin-1 have been demonstrated in SSc^13^, as well as in idiopathic retroperitoneal fibrosis, a disease characterized by abnormal fibrosis of the peritoneum^14^. Elevated levels of eotaxin-1 have been associated with ageing in mice and humans^15^ and an increase in osteoclast activity during periods of bone inflammation^16^.

IL-6 was eight-fold higher in patients with IPF and SSc-ILD (regardless of whether the cutaneous form was limited or diffuse). IL-6 is a pro-inflammatory cytokine that is elevated in many inflammatory and autoimmune conditions, including SSc^17^. It is produced in the smooth muscle cells in the middle tunica layer of blood vessels. Osteoblasts release IL-6 to stimulate osteoclast formation and like eotaxin-1 can be associated with bone inflammation. IL-6 has also been implicated as a mediator of pulmonary fibrosis, regardless of ILD subtype^18^, with IL-6 blockade using tocilizumab reducing the rate of FVC decline in SSc-ILD^19^. Elevated circulating IL-6 has also been associated with IPF acute exacerbations^20^. These and our findings support the presence of potential common pathways for lung fibrosis in SSc-ILD and IPF and suggest the need for a clinical trial of tocilizumab in IPF.

When the IL6 gene was identified as possibly influencing SSc development and progression, analyses identified a substantial association between the minor allele of rs2069840 and limited cutaneous SSc, a trend in association for the minor allele rs1800795 and diffuse cutaneous SSc, and an association with overall SSc and the triplet rs2069840-rs1800795-rs2069827^21^. Following on this result and noting that IL-6 levels are elevated in the sera of SSc patients and are highly correlated with skin damage, led to the suggestion of a clinical trial for tocilizumab, an IL-6 receptor blocker^22^. Our own future research is underway with whole genome sequencing to study the risk of these polymorphisms and potentially more undiscovered IL6 polymorphisms contributing to the risk of developing SSc, with or without ILD, and IPF.

### Cytokines statistically significantly different in SSc

ADAMTS13 was lower in lcSSc without ILD compared to healthy controls in our plasma samples, which is in keeping with previous findings of SSc serum ^23^. ADAMTS13, which cleaves unusually large (and hyperreactive) von Willebrand factor (ULVWF) released from activated endothelial cells to smaller and less active forms, is a metalloproteinase involved in von Willebrand factor coagulation and platelet activation pathways, and is possibly related to the vasculopathy and endotheliopathy present in SSc ^23,24^.

MIG/CXCL9 cytokine levels were substantially elevated only in patients with lcSSc compared to healthy controls regardless of whether ILD was present or absent. The increase in the base-2 log fold change was estimated to be 1.056 (95% CI 0.451, 1.696) for patients with both lcSSc and ILD and 0.707 (95% CI 0.055, 1.359) for patients only with lcSSc. In the log-linear regression model for MIG, the only covariate found to be statistically significant was age (Holm’s *p* = 0.0038) with a base-2 log fold increase of 0.018 (95% CI 0.002, 0.033) per year of age. The effect due to the annual increase in age on MIG/CXCL9 may not seem much, but an additional 56 years of life doubles the level for this cytokine and is sufficient, with our patients with IPF (Table 1) being a couple of decades older than our patients with SSc, to suggest that patients with either IPF or lcSSc have similar increased levels of MIG/CXCL9 if not adjusted for confounding due to age (Fig. 1). MIG/CXCL9 is a proangiogenic chemokine for monocytes leading to immune cell extravasation into tissues and the promotion of pro-fibrotic signaling that has been shown to be upregulated in SSc^25^, as well as a marker for potentially more severe SSc^26^. MIG is also involved in alveolar epithelial cell transition towards a profibrotic phenotype^27^. It is interesting that this study suggests that MIG/CXCL9 levels is a possible biomarker for the milder cutaneous form of SSc. That increased MIG/CXCL9 levels could be a feature of early disease in SSc merits further consideration.

### Cytokines not statistically significantly different between cases and healthy controls

CXCL13 and CXCL10, previously reported to be associated with SSc-ILD and IPF^28^, were not differentially expressed in our patients with either SSc-ILD or IPF compared to healthy controls. This could be related to our relatively small sample sizes or for controlling for multiple testing with Holm’s method. The findings in this study likely identify cytokines with the most drastic differences between healthy and diseased states in peripheral blood, which may potentially have stronger importance in disease mechanisms. Additional SSc related cytokines such as epidermal growth factor and vascular endothelial growth factor were also not significantly different between disease states and healthy controls. Controlling for multiple testing reduces the frequency of false positive findings, but also increases the risk of false negative results. While these findings have not been validated in a replication cohort, previous evidence as cited in the literature strongly reinforces the role of identified cytokines in both SSc-ILD and IPF.

## Conclusion

Differences in circulating cytokines involved in immune cell extravasation and pro-fibrotic signalling provide further evidence of the importance of innate and adaptive immune pathways in the crosstalk with other cellular components leading to pulmonary fibrosis. These peripheral blood findings suggest more widespread systemic activity in ILD disease states and are potential targets for sampling by bronchoscopy to better understand pathogenic mechanisms and to inform drug development. Validation in other patient cohorts and examination of tissue cytokine levels is ongoing as both are required to provide additional insights into the molecular pathways. Further studies of the longitudinal change of these molecules over time are anticipated as these will be more informative for disease progression and response to treatment.

## Supplementary Information

Supplementary Information.

## Data Availability

Anonymized datasets of this study are available from the corresponding author on reasonable request.
