# Supplementary material for: Circulating cytokine levels in systemic sclerosis related interstitial lung disease and idiopathic pulmonary fibrosis
Source: Sci Rep. 2023 Apr 24;13:6647. doi: 10.1038/s41598-023-31232-4 (PMC10125994; doi:10.1038/s41598-023-31232-4)
Supplement: Supplementary file 1 — Supplementary Information. [file 41598_2023_31232_MOESM1_ESM.docx]

**Supplementary Table S1**: List of 87 analyzed cytokines

| **Cytokines** | **Alias** | **Cytokines** | **Alias** | **Cytokines** | **Alias** |
| --- | --- | --- | --- | --- | --- |
| 1. ADAMTS13 |  | 1. IL-2 |  | 1. SAA |  |
| 1. BCA-1 | CXCL13 | 1. IL-20 |  | 1. sCD30 |  |
| 1. CTACK | CCL27 | 1. IL-21 |  | 1. sCD40L |  |
| 1. EGF |  | 1. IL-22 |  | 1. SCF | KIT-ligand |
| 1. ENA-78 | CXCL5 | 1. IL-23 |  | 1. SDF-1alpha+beta | CXCL12 |
| 1. Eotaxin-1 | CCL11 | 1. IL-27 |  | 1. sEGFR |  |
| 1. Eotaxin-2 | CCL24 | 1. IL-28A |  | 1. sgp130 |  |
| 1. Eotaxin-3 | CCL26 | 1. IL-3 |  | 1. sIL-2Ra |  |
| 1. FGF-2 | FGF-β | 1. IL-33 |  | 1. sIL-1RI |  |
| 1. FLT-3L |  | 1. IL-4 |  | 1. sIL-1RII |  |
| 1. Fractalkine | CX3CL1 | 1. IL-5 |  | 1. sIL-4R |  |
| 1. G-CSF |  | 1. IL-6 |  | 1. sIL-6R |  |
| 1. GM-CSF |  | 1. IL-7 |  | 1. sRAGE |  |
| 1. GRO alpha | CXCL1 | 1. IL-8 |  | 1. sTNFRI |  |
| 1. I-309 | CCL1 | 1. IL-9 |  | 1. sTNFRII |  |
| 1. IFN-alpha2 |  | 1. IP-10 | CXCL10 | 1. sVEGFR1 |  |
| 1. IFN-gamma |  | 1. LIF |  | 1. sVEGFR2 |  |
| 1. IL-10 |  | 1. M-CSF |  | 1. sVEGFR3 |  |
| 1. IL-12p40 |  | 1. MCP-1 | CCL2 | 1. TARC | CCL17 |
| 1. IL-12p70 |  | 1. MCP-2 | CCL8 | 1. TGF-alpha |  |
| 1. IL-13 |  | 1. MCP-3 | CCL7 | 1. TNF-alpha |  |
| 1. IL-15 |  | 1. MCP-4 | CCL13 | 1. TNF-beta |  |
| 1. IL-16 |  | 1. MDC | CCL22 | 1. TPO |  |
| 1. IL-17A |  | 1. MIG/CXCL9 | CXCL9 | 1. TRAIL | TNFSF10, CD253 |
| 1. IL-17E | IL-25 | 1. MIP-1alpha | CCL3 | 1. TSLP |  |
| 1. IL-17F |  | 1. MIP-1beta | CCL4 | 1. VEGF-A |  |
| 1. IL-18 |  | 1. MIP-1delta | CCL15 | 1. X6CKine | CCL21 |
| 1. IL-1alpha |  | 1. PDGF-AA |  |  |  |
| 1. IL-1beta |  | 1. PDGF-AB/BB |  |  |  |
| 1. IL-1RA |  | 1. RANTES | CCL5 |  |  |
